# Suzuki Stage VI Unilateral Moyamoya Disease Presenting With Massive Intraventricular Hemorrhage

**DOI:** 10.7759/cureus.55081

**Published:** 2024-02-27

**Authors:** Yudai Hirano, Satoru Miyawaki, Tsukasa Koike, Yasuaki Karasawa, Atsumi Takenobu, Akio Morita, Shotaro Ogawa, Seiei Torazawa, Nobuhito Saito, Akira Teraoka

**Affiliations:** 1 Department of Neurosurgery, University of Tokyo Hospital, Tokyo, JPN; 2 Department of Neurosurgery, The University of Tokyo, Tokyo, JPN; 3 Department of Neurosurgery, Teraoka Memorial Hospital, Fukuyama, JPN

**Keywords:** late stage, suzuki, angiography, intraventricular hemorrhage, choroidal anastomosis, moyamoya disease

## Abstract

Moyamoya disease (MMD) is characterized by stenosis of the terminal portion of the internal carotid artery (ICA) and the development of collateral vessels. In late Suzuki stage MMD, ICA almost disappears, and the moyamoya vessels gradually regress. We report a case of late Suzuki stage unilateral MMD presenting with intraventricular hemorrhage. A 76-year-old woman who had previously been diagnosed with right ICA occlusive disease was referred to our hospital due to impaired consciousness. Radiological evaluation revealed massive intraventricular hemorrhage. After endoscopic hematoma removal, digital subtraction angiography (DSA) was performed to examine the vascular anatomy, which revealed numerous basal moyamoya vessels originating from the posterior cerebral artery. Three-dimensional rotational angiography identified a choroidal anastomosis originating from the posterior choroidal artery as the hemorrhage source. The patient had an *RNF213* p.Arg4810Lys heterozygous variant in the germline. Based on the DSA findings, MMD was diagnosed, and the patient was transferred to a rehabilitation hospital with good postoperative consciousness. In conclusion, patients diagnosed with ICA occlusive disease may have late Suzuki stage MMD, potentially leading to major hemorrhage; therefore, antithrombotic medications should be administered with caution. In diagnosing ICA occlusive disease, the assessment of periventricular anastomosis should be considered, taking into account the possibility of MMD.

## Introduction

Moyamoya disease (MMD) is a progressive disease characterized by the stenosis of the internal carotid artery (ICA) terminus and the development of numerous small collateral blood vessels; its onset can be broadly classified into ischemic and hemorrhagic types [1,2]. MMD is classically divided into six stages according to the Suzuki grade. In stage V and VI Suzuki-grade conditions, the moyamoya vessels regress, and cerebral blood flow depends on the external carotid and vertebral arteries [1]. As Suzuki-grade cases in stages II to IV are the most frequent and those in stages V and VI are rare, the natural history and stroke events in the late Suzuki stage MMD are poorly understood [3,4]. It is difficult to differentiate between the occlusive disease of the ICA and unilateral late-stage MMD using magnetic resonance imaging/angiography (MRI/A). In the guidelines for the diagnosis of MMD, in addition to MRI, digital subtraction angiography (DSA) is recommended for older patients with unilateral disease [5].

The most significant prognostic aggravating factor in MMD is hemorrhagic events. Revascularization surgery for hemorrhagic MMD prevents rebleeding [6]. However, there is no evidence that bypass surgery prevents de novo hemorrhage in patients with asymptomatic MMD. Radiological characteristics associated with hemorrhagic events include choroidal anastomosis and anterior choroidal artery dilatation [7,8]. Revascularization surgery leads to the regression of periventricular anastomosis and peripheral aneurysm [9,10]. The role of choroidal anastomosis in late Suzuki stage MMD, in which moyamoya vessels regress, remains unknown.

Ring finger protein 213 (*RNF213*) was reported as a susceptibility gene for MMD in 2011, and approximately 70% of the Japanese patients diagnosed with MMD harbor the *RNF213 p.Arg4810Lys* variant [11,12]. This variant has been reported to be associated with the age of onset, onset type, and the clinical course of MMD [13]. As the variant is present in about 2% of the general population in East Asian countries [14], it does not directly lead to a definitive diagnosis of MMD. Still, it may be beneficial as an adjunctive diagnosis.

The purpose of the article is to present a case in which the patient was initially diagnosed with ICA occlusive disease at another hospital but later developed massive intraventricular hemorrhage, and a diagnosis of late Suzuki stage MMD (stage VI) was confirmed based on DSA findings.

## Case presentation

**Figure 1:**
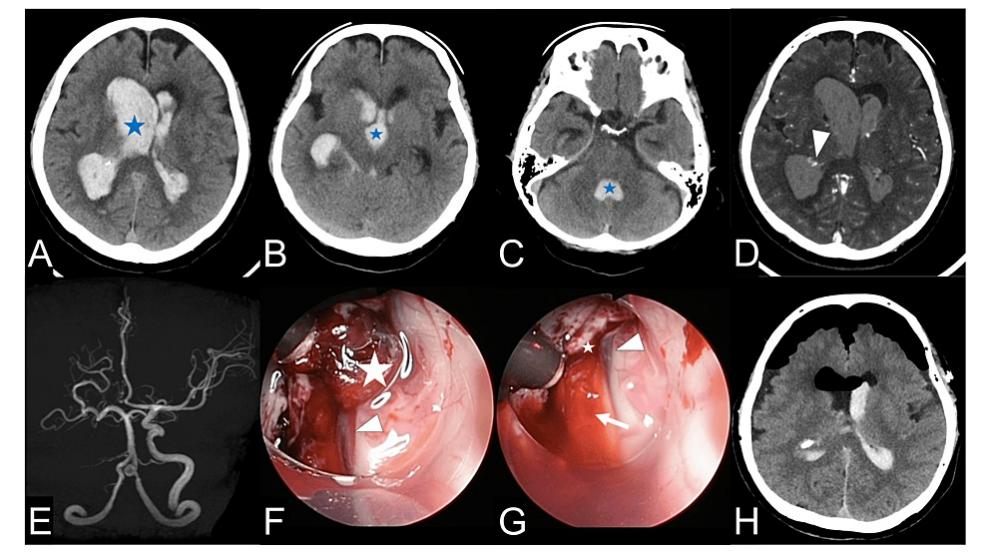
Pre, intra, and postoperative images. A-C: The initial axial non-contrast computed tomography (CT) shows a massive intraventricular hematoma (star) mainly located in the right lateral ventricle (A) extending to the third (B) and fourth ventricle (C). D: Contrast CT shows spot signs (arrowhead) in the posterior horn of the right lateral ventricle, indicating the source of hemorrhage. E: Magnetic resonance angiography shows the occlusion of the right internal carotid artery. F: Intraoperative images in the right lateral ventricles. After removing the hematoma in the right lateral ventricle, the hematoma in the third ventricle (star) was suctioned via the foramen of Monro. The arrowhead indicates the thalamostriate vein. G: Final operative view. The arrowhead indicates the thalamostriate vein, whereas the arrow points to the choroid plexus, and the star indicates the foramen of Monro. H: Postoperative CT shows the sufficient removal of hematoma.

A 76-year-old woman with a sudden impairment of consciousness and a Glasgow Coma Scale score of 6 (E1V1M4) was transported to our hospital for emergency care. She had been previously diagnosed with an occlusive disease of the right ICA at another hospital. The patient had been followed up with conservative treatment with an oral antithrombotic agent to prevent ischemic cerebrovascular events. Her medical history included hypertension and no family history of MMD or stroke. Computed tomography (CT) revealed hematoma packing mainly in the right ventricle and extension to the left, third, and fourth ventricles (Figures 1A-1C). Contrast CT showed a high-spot area in the posterior horn of the right lateral ventricle, which was suspected to be the source of the hemorrhage (Figure 1D). MRA revealed a right ICA occlusion (Figure 1E). Endoscopic hematoma removal was planned immediately to resolve the symptoms caused by the enlarged ventricles. A U-shaped skin incision was made at the right Kocher’s point, and a small two-burr hole craniotomy was performed. A transparent sheath (Neuroport, regular size, outer diameter 10 mm, length 120 mm; Olympus Corporation, Tokyo, Japan) was inserted into the right ventricle, and the hematoma was removed within the visual range; the hematoma in the third ventricle was aspirated from the foramen of Monro and posteriorly removed as far as the endoscope could reach (Figures 1F, 1G). A ventricular draining device was placed, and the operation was completed with sufficient decompression on postoperative CT (Figure 1H).

**Figure 2:**
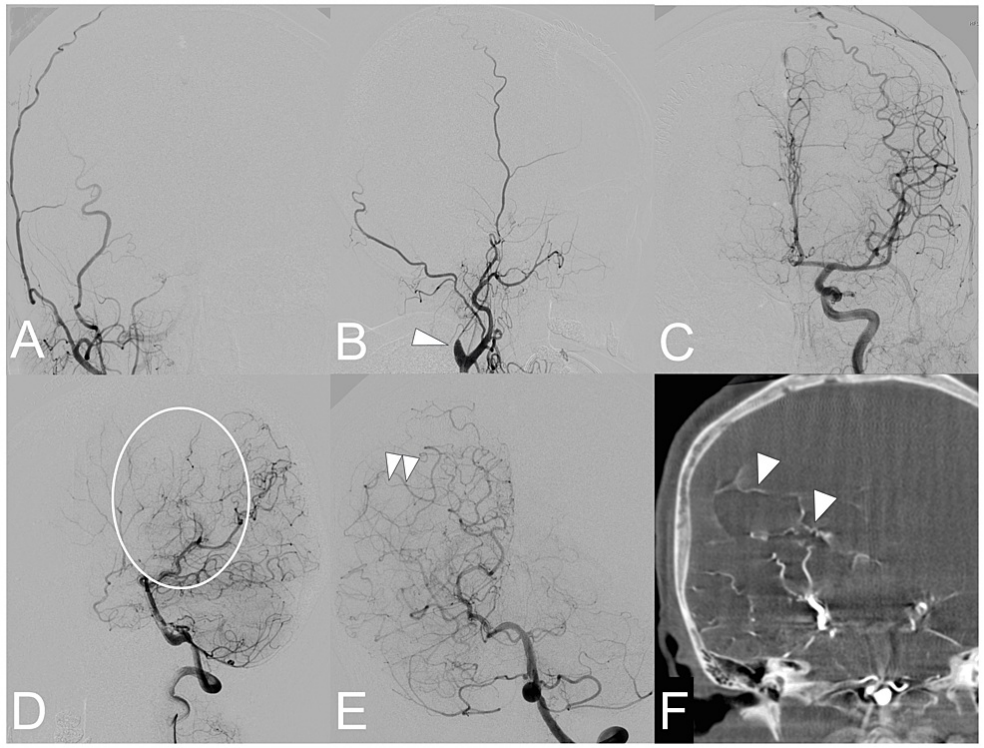
Digital subtraction angiography. A, B: The right common carotid angiography reveals the bottleneck sign (arrowhead) just distal to the cervical bifurcation in the common carotid artery. C: The left common carotid angiography shows no evidence of intracranial stenosis or occlusion. D, E: The lateral view of the vertebral angiography shows the basal moyamoya vessels from the posterior cerebral artery (circle) (D). The anteroposterior view reveals the choroidal anastomosis from the posterior choroidal artery (arrowhead) and no evidence of posterior cerebral artery involvement. F: Three-dimensional rotational angiography reveals the choroidal anastomosis to the medullary artery from the posterior choroidal artery (arrowhead).

DSA was performed postoperatively to examine the vascular structure. The right common carotid angiography showed that the artery was narrowed into a bottleneck shape in the cervical portion, and there was no intracranial regurgitation (Figures 2A, 2B). No transdural anastomosis was identified via the right external carotid artery. The left internal carotid arteriography did not reveal stenosis of the main trunk but demonstrated flow to the bilateral anterior cerebral arteries (Figure 2C). The vertebrobasilar angiography showed abnormal vascular collateral network development at the base of the brain, in addition to normal reflux. There was no stenotic lesion in the posterior cerebral artery, and choroidal anastomosis developed from the posterior choroidal artery (Figure 2E). The three-dimensional rotational angiography confirmed choroidal anastomosis extending to the medullary artery in the right hemisphere (Figure 2F). The patient was diagnosed with Suzuki stage VI unilateral MMD based on the DSA findings.

Genomic DNA was extracted from peripheral blood samples collected from the patient. Direct Sanger sequencing was performed to genotype the *RNF213 p.Arg4810Lys* variant. The specific methods for evaluating the genetic variants of *RNF213 p.Arg4810Lys* followed those previously reported by our group [15]. This study used NM_001256071 and NP_00124300 from the National Center for Biotechnology Information as reference sequences. The ethical review of the genetic analysis was approved by the Human Genome Gene Analysis Research Ethics Committee of the Faculty of Medicine, University of Tokyo (approval number: G10026) and by the Ethics Committee of the Teraoka Memorial Hospital (approval number: FY2015-01). As the patient herself had an altered mental status, informed consent was obtained from the husband and daughter.

## Discussion

We report a case of late Suzuki stage unilateral MMD presenting with massive intraventricular hemorrhage with an *RNF213 p.Arg4810Lys* heterozygous variant. The patient was initially diagnosed with ICA occlusive disease and was prescribed antithrombotic drugs to prevent ischemic events. However, choroidal anastomosis from the posterior choroidal artery had developed, leading to hemorrhage. DSA was necessary to differentiate between ICA occlusive disease and late Suzuki stage MMD. Late Suzuki stage MMD with Suzuki stages V-VI is infrequent, accounting for approximately 10% of all MMD cases, and its natural history is unknown [3,4]. Higher Suzuki stage IV-VI MMD is associated with ethmoidal and vault collaterals, and the onset type has been suggested to be associated with cerebral hemorrhage [3]. The angiographic Suzuki stage tends to be more advanced in hemorrhagic patients than in ischemic patients [4].

Because ICA occlusive disease can cause cerebral infarction, medical therapy with antiplatelet agents is indicated in symptomatic cases [16]. However, in the presence of a background MMD, as in the present case, the prescription of antithrombotic drugs should be carefully considered, especially when angiographic features indicate a high risk of hemorrhage. Studies on the safety of antiplatelet agents for MMD are limited, and cilostazol can potentially reduce mortality compared with other antiplatelet agents [17]. However, the small number of studies and lack of randomized trials have led to uncertainty regarding the benefits and risks of these agents. Whether the use of antithrombotic agents increases the risk of hemorrhage, especially in the setting of high-risk radiological findings of hemorrhage, is a subject for future studies.

Guidelines for diagnosing MMD suggest that MRI/A alone should be used only in bilateral cases, whereas DSA is recommended in unilateral cases or older patients to differentiate MMD from other diseases [5]. MRA and CT angiography cannot distinguish late Suzuki stage MMD from ICA occlusive disease. As in this case, DSA was useful in accurately evaluating the development of choroidal anastomosis, a characteristic of MMD and a high-risk structure for hemorrhage. Recently, the usefulness of MRA coronal images reformatted as sliding thin slab maximum intensity projection coronal images in assessing periventricular anastomosis has also been reported [18], and combining these multiple modalities may lead to an accurate diagnosis and prognostic prediction. Genetic testing using the disease susceptibility gene *RNF213* is helpful as an adjunct diagnostic tool for MMD, in which a heterozygous variant of *RNF213 p.R4810K*, the most frequent variant, was identified. However, as about 2% of the general population in East Asian countries has the same variant [14], genetic testing alone cannot lead to a definitive diagnosis. Further studies are required to introduce genetic variants into the diagnostic criteria of MMD.

In hemorrhagic MMD, rebleeding is a factor that significantly worsens the prognosis, and its prevention is a primary concern. The Japanese Adult Moyamoya trial, a multicenter, prospective, randomized controlled trial, demonstrated that bypass surgery effectively prevented rebleeding in patients with specific backgrounds [6,19]. The inclusion criteria for the study included age between 16 and 65 years and a modified Rankin scale score of 0 to 2, indicating independence. On the other hand, the effect of revascularization surgery to prevent rebleeding in patients with severe neurological symptoms due to massive initial hemorrhage, with a modified Rankin scale score of 3 or higher, or in patients older than 65 years, is uncertain. Based on the patient’s advanced age and poor modified Rankin scale score, the decision was not to perform the bypass surgery.

In the present case, asymptomatic MMD caused de novo hemorrhage in advanced age. Several factors have been reported to be involved in the hemorrhage of MMD, particularly the radiological features of the vascular structures associated with hemorrhage-onset MMD. Thalamic and choroidal periventricular anastomoses have been associated with hemorrhagic MMD [8]. Choroidal anastomosis has also been associated with de novo hemorrhage in the non-hemorrhagic hemisphere in patients with hemorrhagic MMD [20]. Periventricular anastomoses have been radiologically documented to regress after bypass surgery [10]. Despite these reports on factors associated with hemorrhagic MMD, there are no publications or randomized controlled studies of surgical intervention leading to hemorrhage prevention in asymptomatic patients with MMD. The future challenge is implementing bleeding against prophylaxis in patients with high-risk vascular structures, and the results of future studies are anticipated.

## Conclusions

Patients diagnosed with ICA occlusive disease may have late Suzuki stage MMD, which can cause major hemorrhage; therefore, antithrombotic medications should be administered with caution. Late Suzuki stage MMD, in which the moyamoya vessels regress, may also present with hemorrhagic events associated with the development of choroidal anastomosis. Thus, in diagnosing ICA occlusive disease, the assessment of periventricular anastomosis should be considered, taking into account the possibility of MMD. Whether prophylactic bypass surgery for asymptomatic MMD patients should be indicated in the presence of high-risk structures for hemorrhage requires future studies.
